# ^125^I Seed Promotes Apoptosis in Non-small Lung Cancer Cells *via* the p38 MAPK-MDM2-p53 Signaling Pathway

**DOI:** 10.3389/fonc.2021.582511

**Published:** 2021-04-21

**Authors:** Tao Zhang, ZhiQiang Mo, Guangfeng Duan, Rijie Tang, Fujun Zhang, Mingjian Lu

**Affiliations:** ^1^Department of Radiology, Affiliated Cancer Hospital & Institute of Guangzhou Medical University, Guangzhou, China; ^2^Department of Interventional Radiology, Guangdong Provinical People's Hospital, Guangzhou, China; ^3^Department of Medical Imaging & Interventional Radiology, Cancer Center and State Key Laboratory of Oncology in South China, Sun Yat-sen University, Guangzhou, China

**Keywords:** ^125^I seed, non-small cell lung cancer, p38, p53, MDM2

## Abstract

**Purpose:** ^125^I seeds were effective in the treatment of non-small cell lung cancer in previous research. However, the exact signaling pathway-mediated apoptosis mechanism is still unclear. The present study analyzed the effects and potential mechanisms of ^125^I seed on the growth and migration of A549 cells.

**Methods:** Lung cancer A549 cells were irradiated with ^125^I seed for various times. MTT, invasion assay, and flow cytometry were used to detect the proliferation, invasion, and apoptosis of treated cells, respectively. A Nimblegen genome-wide expression profile chip was used to evaluate gene expression changes in ^125^I seed-treated A549 cells. Validation studies were performed using phosphorylated protein chip technology, Western blot, nude mouse tumor xenograft assay, and immunohistochemical experiments. All statistical analyses were performed using unpaired Student's *t* tests and Kruskal-Wallis test.

**Results:** Irradiation with ^125^I seed inhibited A549 cell proliferation and invasion and induced apoptosis (primarily early apoptosis). Irradiation with ^125^I seed also caused the downregulation of p38MAPK, degradation of mouse double-minute 2 homolog (MDM2), and higher expression of p53, which eventually resulted in non-small cell lung cancer cell apoptosis.

**Conclusion:** ^125^I seed irradiation activated the p38MAPK/MDM2/p53 signaling pathway and promoted non-small cell lung cancer cell apoptosis. Future clinical studies targeting this signal may provide a new potential therapeutic approach for non-small cell lung cancer.

## Introduction

^125^I seed implantation is a low-dose-rate brachytherapy, and it was successfully used in prostate cancer with few complications (1). Recent studies confirmed that ^125^I seed brachytherapy obtained favorable local control efficacy in other solid tumors, such as lung cancer, liver cancer, metastatic malignant melanoma, and pancreatic cancer (2–6). A total of 1.3–1.5 million people are diagnosed with lung cancer annually worldwide, and non-small cell lung cancer (NSCLC) accounts for 80% (7, 8). Most NSCLC is stage IIIB–IV at the time of initial diagnosis in China. Chemotherapy remains the mainstay of treatment for advanced stage IIIB–IV NSCLC (9). Combined therapy, such as chemoradiotherapy, is preferred for patients with good performance status, and it achieves additive or synergistic therapeutic effects. Combined treatment increases the occurrence and severity of adverse events, such as bone marrow suppression, gastrointestinal reactions, radioactive pneumonia, and radioactive esophagitis (10). These side effects reduce the life quality and treatment compliance of patients. When external irradiation was performed in NSCLC, an intensive dose is often needed to achieve better local control, which may increase the damage to normal organs and tissues around the tumor.

Chemotherapy combined with ^125^I seed implantation in locally advanced lung cancer improved localized control in patients compared with chemotherapy alone (11). Notably, this strategy did not significantly increase the complications or toxicity of combined therapy, and it improved the patients' quality of life. Previous studies revealed the potential antitumor mechanisms of ^125^I seed brachytherapy, including cell cycle arrest, inhibition of proliferation, induction of cell apoptosis, effects on cell signal transduction, and inhibition of tumor angiogenesis (12, 13). However, due to the inherent complexity of the molecular mechanism in tumor therapy, numerous problems are worth further investigation.

The present study examined the effects and underlying molecular mechanisms of ^125^I seed on NSCLC cells. To the best of our knowledge, this is the first study to use a Nimblegen genome-wide expression profile chip to evaluate gene expression changes in ^125^I seed-treated A549 cells.

## Materials and Methods

### Cell Culture

The cell line A549 was from State Key Laboratory of Oncology in Southern China, Sun Yat-sen University, Guangzhou, P. R. China. The A549 human lung carcinoma cell line was cultured in DMEM supplemented with heat-inactivated fetal calf serum (Gibco, Shanghai, China), penicillin (100 U/ml), and streptomycin (100 mg/ml) in 5% CO_2_ at 37°C.

### Chemicals and Antibodies

A Cell Proliferation Kit I (MTT) was purchased from Roche Diagnostics GmbH (Mannheim, Germany). The antibodies used were anti-p38MAPK (14451, Cell Signal Technology, USA), antiphosphorylated p38MAPK (4511, Cell Signal Technology, USA), anti-MDM2 (86934, Cell Signal Technology, USA), antiphosphorylated MDM2 (3521, Cell Signal Technology, USA), anti-p53 (2527, Cell Signal Technology, USA), antiphosphorylated p53 (82530, Cell Signal Technology, USA), antiphosphorylated ATM (13050, Cell Signal Technology, USA), anti-ATM (2873, Cell Signal Technology, USA), antiphosphorylated H2AX (9718, Cell Signal Technology, USA), anti-H2AX (7631, Cell Signal Technology, USA), cleaved-caspase3 (9664, Cell Signal Technology, USA), and caspase3 (14220, Cell Signal Technology, USA). SB203580 and NSC207895 were purchased from Selleckchem (Houston, USA). β-Actin antisense oligonucleotides were purchased from Invitrogen (New York, USA). Human CGH4x72K Whole-Genome Tiling Array was purchase from Roche-NimbleGen (New York, USA). Proteome Profiler Human Phospho-MAPK Array Kit was purchased from R&D (Minneapolis, USA). Fluorescein isothiocyanate 4,6-diamidino-2-phenylindole (DAPI), diaminobenzidine (DAB) detection, and secondary antibody conjugated with horseradish peroxidase (HRP) were obtained from Zsbio (Beijing, China).

### ^125^I Seed and ^125^I Seed Irradiation Model

^125^I seeds (6711/BT-^125^I) were manufactured by Beijing Atom and High Technology Industries, Inc. (Beijing, China). The radiation activity of each seed was 0.63–0.81 mCi. Each seed was examined for sealing and activity before use. The dose distribution of ^125^I seeds in experiments was calculated using the treatment planning system (TPS) and proven homogeneous distribution. A 96-well plate transwell installation system was used to investigate the effects of ^125^I seeds on cell proliferation. For the investigation of cell motility, a 24-well plate transwell installation was used. For Western blot assays, a six-well plate transwell installation system was used to obtain sufficient protein. The irradiation distance of the cells was ~3 mm (14).

### MTT Assay

A549 cells were seeded in a 96-well plate at a density of 5,000 cells per well and incubated until cell attachment. Six plates were irradiated by ^125^I seed for 12, 24, 36, 48, 72, or 96 h. A control plate was cultured for the same time without irradiation. A 0.5% MTT solution (20 μl) was added to each well and incubated at 37°C for 6 h. DMSO (200 μl) was added to each well to dissolve the formazan product. After shaking for 30 min, the absorbance was measured at 570 nm using a multiwell plate reader.

### Flow Cytometric Analysis

Cell apoptosis was evaluated using flow cytometry. For the ^125^I seed irradiation group, A549 cells were seeded in a six-well plate and irradiated with seven ^125^I seeds for 24, 48, and 72 h. Cells were trypsinized, centrifuged at 1,200 rpm for 5 min, and washed twice in cold PBS sequentially before being resuspended in 0.4 ml binding buffer. Cells were incubated in the dark for 15 min after 5 μl Annexin V-FITC was added in suspension. Cells were incubated for another 10 min after the addition of propidium iodide (10 μl) in suspension. The samples were analyzed using a flow cytometer (Beckman Coulter, Brea, USA).

### Transwell Assay

Cell migration assays were performed in 24-well plates using the transwell system (Corning, NY, USA), which allows cells to migrate through a polycarbonate membrane with an 8-μm pore. Two ^125^I seeds were placed in the bottom of the well, and A549 cells were seeded on the chamber. ATP (100 μM) was included or omitted in the medium in the upper and lower compartments of the chambers. The plates were incubated at 37°C for the indicated time periods. The membranes were washed with PBS, followed by cell fixation in cold methanol for 15 min. The cells were stained with crystal violet. Cells above the membrane were gently removed using a cotton swab. Cells beneath the membrane were counted in five microscopic fields. Each experiment was performed using three transwell chambers and repeated three times.

### Western Blot Analysis

A549 cells were irradiated with ^125^I seed for 72 h before harvesting and lysing for protein extraction. The protein concentration was determined using the Bio-Rad protein assay kit (Bio-Rad, Shanghai, China). Lysates were separated using electrophoresis and transferred to PVDF membranes. The membranes were blocked in 5% non-fat dry milk dissolved in 0.1% Tween 20 Tris-buffered saline (TBST) at room temperature for 1 h and incubated with primary antibodies (dilution ratio, 1:1,000) at 4°C overnight. After three washes with TBST, membranes were incubated with a horseradish peroxidase-conjugated antirabbit IgG secondary antibody (dilution ratio, 1:2,000) for 2 h at room temperature. The blots were visualized using an enhanced chemiluminescence detection system (Millipore, Billerica, USA). Beta-actin was used as a loading control. ImageJ was used to analyze the band density of Western blot strips.

### Gene Data Expression Analysis

RNA was isolated from A549 cells after irradiation with ^125^I seeds for 72 h using TRIzol reagent and purified using a PureLink™ RNA Mini Kit (Ambion, USA). RNA was quantified using spectrophotometry, and the measurement of the absorbance values at 260 and 280 nm, and the 260/280 nm ratio was used to estimate the level of protein contamination. The 260/280 nm ratios of the samples ranged from 1.8 to 2.0. The extracted total RNA from A549 cells was used for cDNA synthesis. The labeled cDNA was purified and hybridized to the microarray, and the arrays were washed and stained following the manufacturer's instructions. Global gene expression was analyzed using a Human CGH 4 × 72K Whole-Genome Tiling Array (Roche-NimbleGen, USA), and microarray experiments were performed by KangChen Biotech (KangChen, Shanghai, China). Data were obtained using the Agilent Feature Extraction software. GO analysis and pathway analysis were performed on this subset of genes. The functions of extracted genes were sorted based on the analytical program Database for Annotation Visualization and Integrated Discovery.

### Human Phospho-MAPK Array

A549 cells were collected after irradiation with 125I seeds for 72 h and lysed in cell lysis buffer (200 μl). Array Buffer 1 (Proteome Profiler Human Phospho-MAPK Array Kit, R&D, USA) was used to dilute the sample to 1.5 ml. Each film used 2 ml of Array Buffer 5 for closed processing, and films were incubated for 1 h on a shaker. The sample was dissolved in 20 μl of a detection antibody complex, blended, and incubated for 1 h at room temperature. Prepared samples were combined, sealed for fluid absorption, and incubated 12 to 14 h at 4°C. The membrane was washed with 20 ml of 1 × wash buffer three times for 10 min each. A streptavidin–HRP solution was prepared and diluted in Array Buffer 5. Each film required 2 ml of corresponding diluent and was incubated 0.5 h at room temperature. The membrane was washed with 20 ml of 1 × wash buffer three times, for 10 min each. Membranes were incubated in 1 ml of chemical chromogenic reagent, and the air bubbles were minimized as much as possible during the 1-min incubation. Residual chromogenic reagent was absorbed, and the chromogenic reaction was performed in a darkroom cassette for 10 min.

### A549 Cell Tumor Mouse Model

Thirty female BALB/C nude mice (Inbred Mice, Medical Experimental Animal Center of Guangdong Province, SPF) at 4 weeks old were used for the *in vivo* study. A549 cells were injected (0.2 ml per mouse, concentration of 1 × 10^7^/ml) on the medial side of the thigh. After ~2 weeks, the tumor diameter was ~1 cm. Nude mice with similar tumor sizes were selected for subsequent study, and mice without tumors or tumors that were too small (<0.5 cm, *n* = 4) or too large (more than 1.5 cm, *n* = 5) were eliminated. Tumors with a diameter <0.5 cm were not conducive to implantation of ^125^I seed, and the tumor burden was too large for nude mice with tumor diameters >1.5 cm. Twenty-one nude mice were selected and randomly divided into three groups: seed group A, control group B (0.8 mCi ^125^I seed), and non-activity seed group C (^125^I seed with zero activity). Each mouse in groups B and C were implanted with only one ^125^I seed. An 18-gauge needle was gradually inserted into the tumor, and a turntable implantation gun was used to implant the seed into the tumor. Over the next 10 days, we determined the tumor volumes. The length (*a*, cm) and width (*b*, cm) of each tumor were measured using a Vernier caliper every 2 days, and tumor volumes were calculated according to the following formula *V* = (*a* × *b*^2^)/2. All mice were euthanatized using carbon dioxide. (The nude mice had no heartbeat or breathing). Tumors were extracted and placed in 10% formalin waiting for pathological examination. The Sun Yat-sen University Ethics Committees approved this study, which abided ethical guidelines.

### Immunohistochemical Analysis

Briefly, paraffin-fixed tumor tissue was sliced, and the sections were deparaffinized and hydrated. Citrate buffer (0.1 M, pH 6.0) was heated to boiling in a microwave oven, and the sections were placed in the buffer for 10 min for antigen retrieval. PBS was used to wash the residual liquid twice, and the slices were blocked with 1% bovine serum albumin for 10 min. The sections were incubated with rabbit antihuman p-p38, p-p53, and p-MDM2 (Cell Signaling Technology, USA) antibodies (1:100) overnight at 48°C and incubated with goat antirabbit Envision for 30 min at room temperature. Negative controls were treated with PBS rather than rabbit antihuman antibodies under the same conditions. The chromogenic substrate 3,30-diaminobenzidine tetrachloride (DAB; Dako, Copenhagen, Denmark) was added for 30 s. Image Pro Plus 6.0 (Media Cybernetics, Rockville, MD) was used to calculate the optical density of p-p38, p-p53, and p-MDM2.

### Statistical Analysis

All statistical analyses were performed using SPSS 20.0 (SPSS Inc., Chicago, IL). Differences in mean values between two groups were analyzed using unpaired *t* tests, and the means of more than two groups were compared using the Kruskal-Wallis test. Dunnett's multiple comparison test was used for *post hoc* testing. *P* < 0.05 was considered statistically significant.

## Results

### Effects of ^125^I Seed Brachytherapy on the Proliferation and Migration of A549 Cells

**Figure 1:**
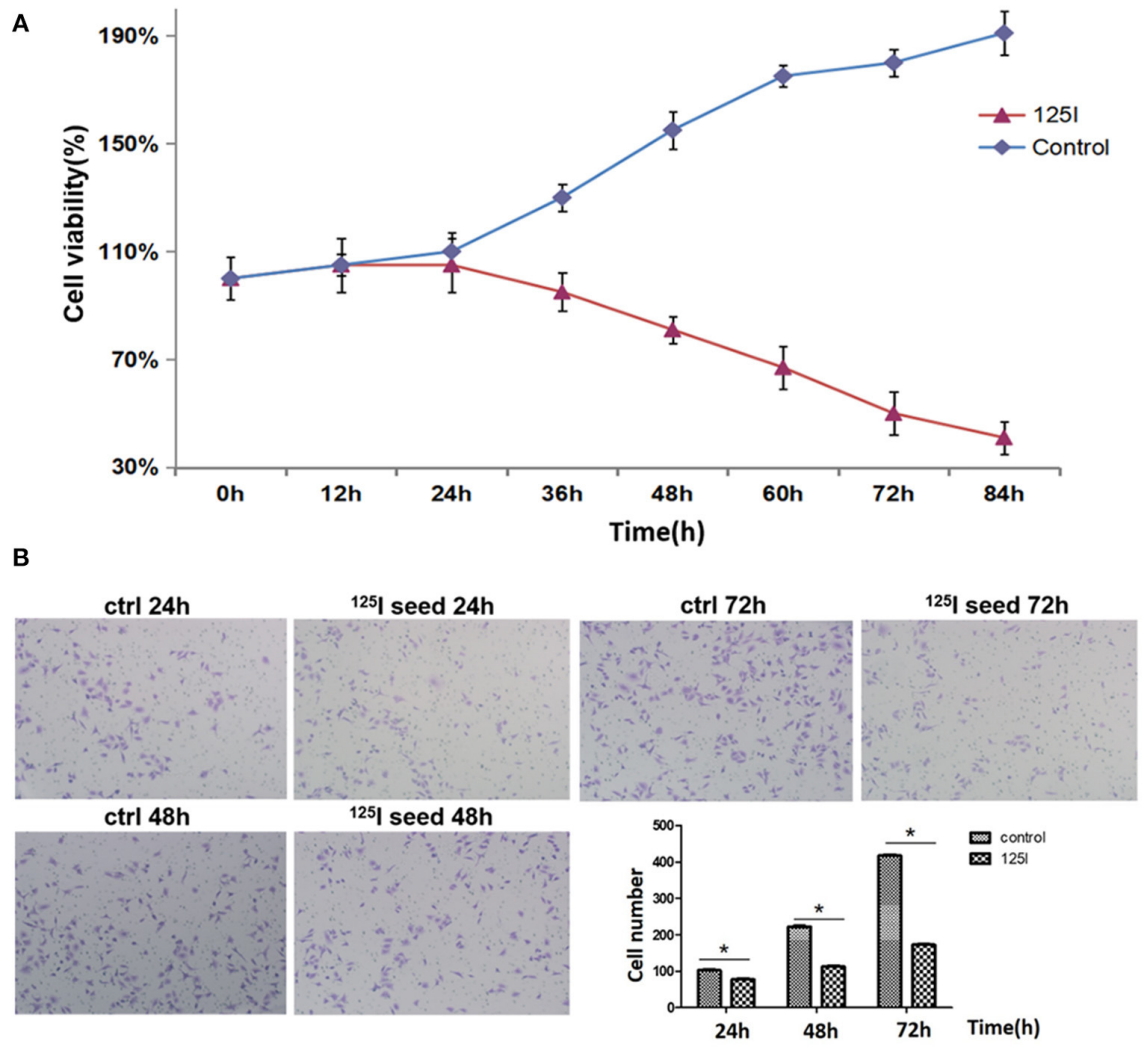
**(A)** MTT analysis indicates that ^125^I seed treatment gradually suppresses the proliferation of A549 cells. These data are representative of three independent experiments. **(B)** The transwell assay showed that the ^125^I seeds gradually inhibited the migration of A549 cells over time. These data represent the means ± SD of three independent experiments. The differences between the control and treated groups were statistically significant (^*^P < 0.05).

We first examined A549 cell proliferation after ^125^I seed irradiation for various periods of time. MTT assays showed that the proliferation of A549 cells declined after 24 h of irradiation (Figure 1A). The cell proliferation rate gradually declined with the increase in irradiation time. A549 cell proliferation was reduced by 50% at 72 h. Transwell chamber migration experiments showed that the migration ability of A549 cells gradually decreased with increasing irradiation time of the ^125^I seed (Figure 1B). A slight decrease in cell migration ability was observed in ^125^I seed-irradiated cells at 24 h compared with controls (102.8 ± 11.8 vs. 120.3 ± 12.3 cells, respectively), and this trend remained at 48 h (234.4 ± 14.6 vs. 138.6 ± 17.5 cells) and 72 h (418.3 ± 20.6 vs. 221.5 ± 18.6 cells) (*P* < 0.05).

### Effects of ^125^I Seed Brachytherapy on the Apoptosis of A549 Cells

**Figure 2:**
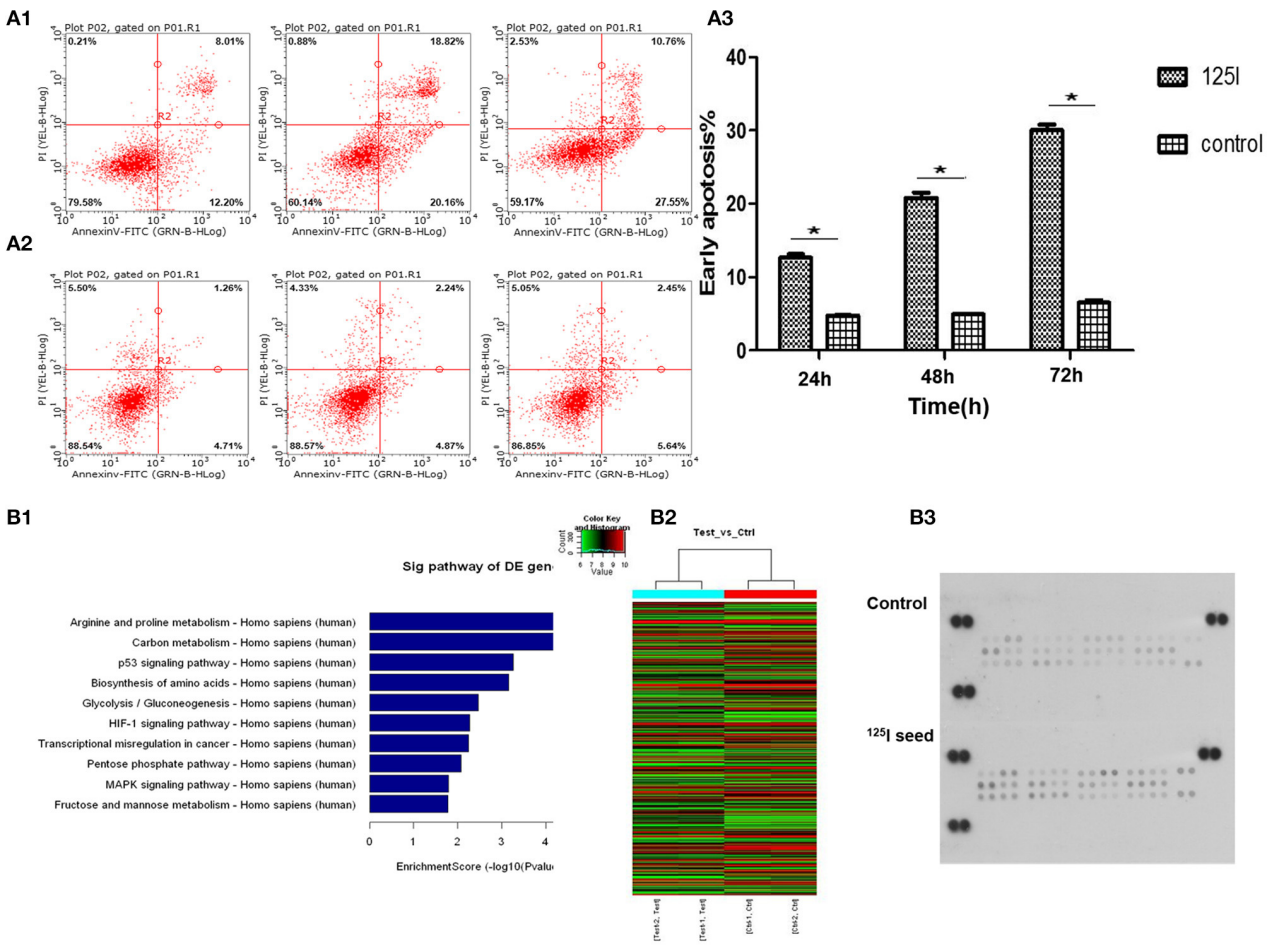
Flow cytometric analysis indicates that the treatment of ^125^I seed for different times induces early apoptosis in A549 cells. **(A1)** With the increase in irradiation time, the early apoptosis in the ^125^I seed group increased gradually. **(A2)** The early apoptosis of control group cells had no obvious change. **(A3)** These data represent the means ± SD of three independent experiments. The difference between control and treated groups is statistically significant (*P < 0.05). The gene chip screened out 10 significant signal pathways **(B1)**. The results of the heat map of gene detection **(B2)**. Human phospho-MAPK array shows that ^125^I seed activated (phosphorylated) MAPK family members Erk1/2, JNK (1–3), and p38 (α/β/δ/γ), and the Akt, GSK-3, p70 S6 kinase, TOR, p53, and CREB **(B3)**.

A549 cells were exposed to 0.8 mCi activity of type 6711 ^125^I seed with an initial dose rate of ~0.96 cGy/h for 24, 48, and 72 h, and the irradiation doses were 1.61, 3.22, and 4.84 Gy, respectively. A gradual increase in the apoptosis ratio (early + late) was observed in the irradiation group compared with the control group in a dose- and time-dependent manner (Figure 2A1–3). Notably, a significant increase in early apoptosis was observed in the ^125^I seed irradiation group at 24, 48, and 72 h (12.2% ± 1.16, 20.16% ± 1.78, and 30.77% ± 1.21, respectively, compared with 4.82% ± 1.06, 4.97% ± 1.52, and 5.24% ± 1.33 in the control group) (*P* < 0.05).

**Figure 3:**
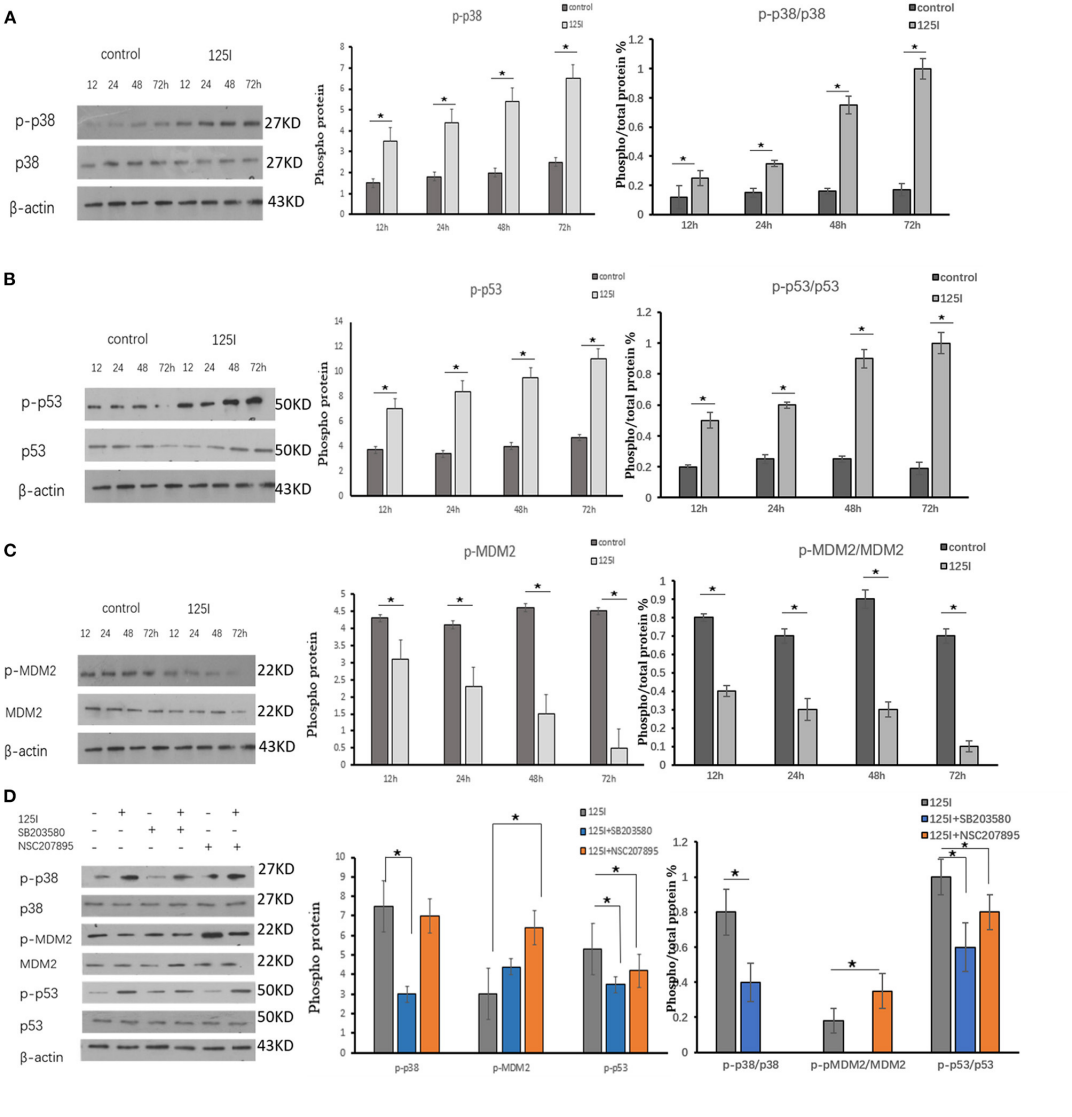
**(A–C)** A549 cells were irradiated in six-well plates for 12, 24, 48, and 72 h. Compared with the control group, the intracellular phosphorylation levels of p38 and p53 increased gradually over time in the ^125^I seed group, and MDM2 was reduced over time. P38, p53, and MDM2 total protein expression was not different between the two groups. The ratio of phosphorylated p38 and p53 protein to total protein increased over time, and it decreased MDM2. The data are presented as the means ± SD. **(D)** The expression of p-p38, p-pMDM2, and p-p53 and their total proteins were analyzed using Western blot analysis. After irradiation by ^125^I seed, p-p38 and p-p53 expression levels increased and p-MDM2 expression level decreased. The p38 inhibitor (SB203580) suppressed the expression level of p-p38, increased the expression levels of p-MDM2, and decreased the expression levels of p-p53. The level of p38, MDM2, and p53 showed no obvious changes. The data are represented as the means ± SD of three independent experiments. There was statistical significance between the control and treated groups, *P < 0.05.

### Gene Chip Screening Selected the MAPK Signaling Pathway and p53 Signaling Pathway

We performed gene array analysis in A549 cells with/without ^125^I seed irradiation. The exposure time was 72 h for irradiated cells. Nimblegen whole genome expression microarray analysis detected 33 differentially activated signal pathways and 622 differentially expressed genes in irradiation cells compared with the control cells. Gene Ontology (GO) and pathway analysis were performed on the identified genes, and the MAPK pathway, p53 pathway (upregulated), and MDM2 gene (downregulated) were screened using our abundance of integral (enrichment score) for further study (Figure 2B1). The heat map of gene detection is shown in Figure 2B2.

### Human Phospho-MAPK Array Analysis

MAPK protein microarray analysis was performed to identify activated (phosphorylated) proteins in A549 cells after irradiation with ^125^I seeds for 72 h compared with the control cells. The results showed that activated (phosphorylated) MAPK family members Erk1/2, JNK (1–3), and p38 (α/β/δ/γ), and the Akt, GSK-3, p70 S6 kinase, TOR, p53, and CREB were potentially involved in the signal transduction, proliferation, and apoptosis in irradiated cells. Quantification of the results showed that phospho-p38 and phospho-p53 levels were the most significantly increased factors of all of the identified proteins (Figure 2B3, *P* < 0.05). Therefore, we focused our subsequent experiments on MDM2 (identified in the gene array) and p38MAPK and p53 (identified in the MAPK array analysis).

### Western Blot Analysis of p-p38MAPK-, p-MDM2-, and p-p53in-Irradiated A549 Cells

Western blot analysis was performed to verify activation of p38MAPK, MDM2, and p53 in A549 cells irradiated for 12, 24, 48, and 72 h. The intracellular phosphorylation levels of p38 and p53 increased gradually in the ^125^I seed group over time, and the levels of MDM2 were reduced. The total protein expression of p38, p53, and MDM2 was not different between the two groups. The ratio of phosphorylated p38 and p53 protein/total protein increased over time in the ^125^I seed group and MDM2 decreased. The results confirmed that p-p38MAPK and p-p53 were upregulated in irradiated cells, but p-MDM2 was downregulated, compared with those in the control cells (Figures 3A–C).

### Western Blot Analysis and Flow Cytometry Results of a p38MAPK Inhibitor and MDM2 Agonist on ^125^I Seed-Induced Apoptosis of A549 Cells

**Figure 4:**
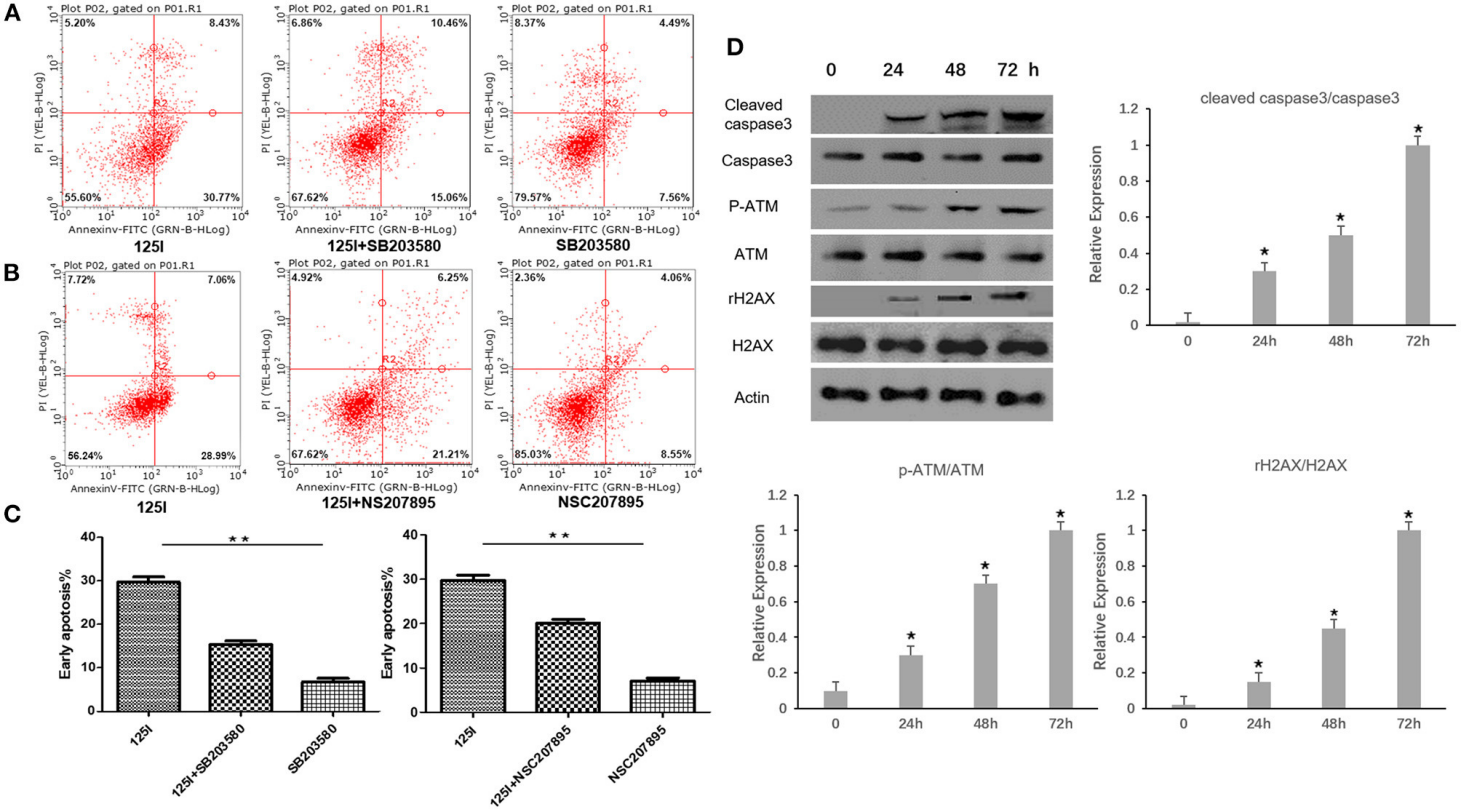
Flow cytometric analysis after treatment of ^125^I seed for 72 h. **(A)** The early apoptosis rate of the ^125^I seed group (left), the ^125^I+SB203580 group (middle), and the SB203580 group (right) is 30.77% ± 1.21, 15.06% ± 1.73, and 7.06% ± 1.54, respectively; **P < 0.01. **(B)** The early apoptosis rate of the ^125^I seed group (left),the ^125^I+NSC207895 group (middle), the NSC207895 group (right) is 28.99% ± 1.15, 21.21% ± 1.52, and 8.55% ± 1.27, respectively; **P < 0.01. The data represent the means ± SD of three independent experiments. **(C)** The difference between each groups is statistically significant (**P < 0.01). **(D)** The level of cleaved-caspase3, p-ATM, and γ-H2AX increased gradually with the increase of irradiation time. *P < 0.05.

The p38 inhibitor SB203580 (Selleckchem, Houston, USA) and the MDM2 agonist NSC207895 (Selleckchem, Houston, USA) were used to determine the functional contribution of these factors in the apoptosis of ^125^I seed-irradiated A549 cells. After irradiation, p-p38 and p-p53 expression levels increased, and p-MDM2 expression level decreased. The p38 inhibitor SB203580 suppressed p-p38 expression, increased p-MDM2 expression levels, and decreased p-p53 expression levels. The ^125^I +SB203580 group and ^125^I+NSC207895 group had lower early apoptosis than the ^125^I seed alone group (Figures 3D, 4A–C). Irradiation with ^125^I seed caused a downregulation of p38MAPK, MDM2 degradation, and higher p53 expression.

### Western Blot Analysis of Cleaved-Caspase3, p-ATM, γ-H2AX in Irradiated A549 Cells

Western blot analysis was performed in A549 cells after being irradiated for 24, 48, and 72 h. The level of p-ATM and γ-H2AX related to gene damage increased gradually with the increase of irradiation time. At the same time, the cleaved-caspase3 was found to increase gradually with the increase of irradiation time (Figure 4D).

### Effects of ^125^I Seeds on A549 Cells *in vivo*

**Figure 5:**
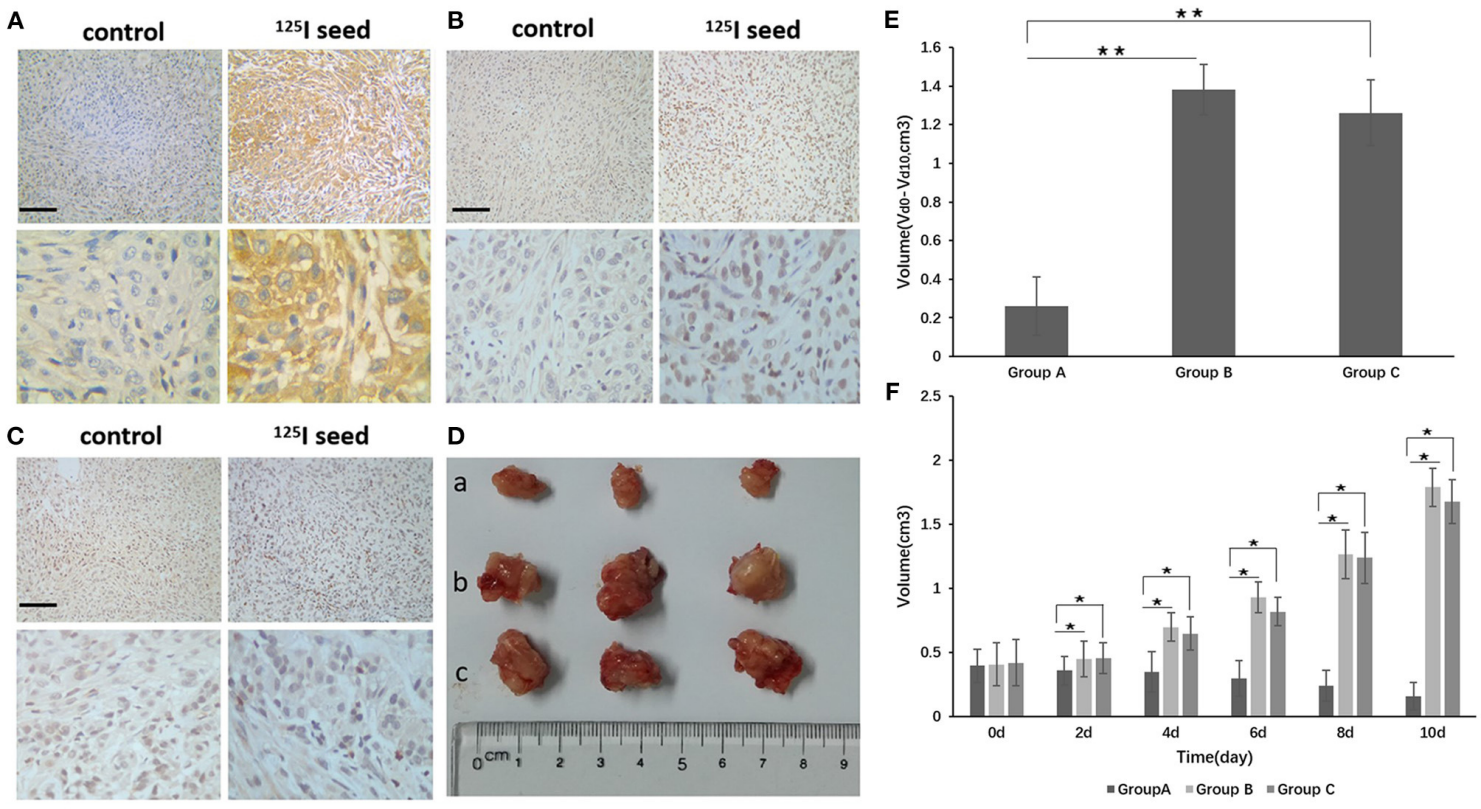
**(D)** After implantation of the ^125^I seed for 10 days, the tumor was removed and the tumor was measured (a group: ^125^I seed group, b group: control group, c group: non-activity ^125^I seed group). **(E)** The Vd0-d10 of group A was obviously reduced compared with groups B and C. *Vd0-d10*: The change in volume between d0 and d10. **(F)** There were differences in the volume changes between groups A and B and between groups A and C at 2, 4, 6, 8, and 10 days. The differences between each group are statistically significant (**P < 0.01). Tumor tissue immunohistochemistry found that p-p38 and p-p53, and the apoptotic body expression were upregulated in the ^125^I seed group, but p-MDM2 expression was downregulated compared with the control group and the non-activity seed group [**(A)** p-p38, **(B)** p-p53, **(C)** p-MDM2]. The original magnification is 200. The bar length represents 50 μm. *P < 0.05.

We established an A549 cell tumor mouse model and examined three groups: seed group A, control group B (0.8 mci ^125^I seed), and non-activity seed group C (^125^I seed with no activity). After 10 days of observation, we found that the tumor growth of group A was obviously inhibited compared with groups B and C. The Vd0-d10 of group A was obviously inhibited compared with groups B and C. There were differences in the volume changes between groups A and B and between groups A and C at 2, 4, 6, 8, and 10 days. The difference between each groups was statistically significant (Figures 5D–F), and the inhibitory effect was statistically significant (*P* < 0.05). No statistically significant difference was observed in group A compared with group C.

### Immunohistochemical Analysis of p-p38, p-p53, and p-MDM2 Expression

The mice were killed, and the tumor tissue was removed and subjected to immunohistochemistry. The positive expression rate of p-p38 in tumor tissues from group A (^125^I seed treatment) was significantly higher than group B (control) and group C (non-activity ^125^I seed) [85.7% (12/14) compared with 28.5% (4/14) and 35.7% (5/14), respectively] (*P* <0.05, group A vs. B) (Figure 5A). The positive expression rate of p-p53 showed a similar trend. Group A (^125^I seed) showed a p-p53-positive rate of 71.4% (10/14) compared with group B (control) at 14.2% (2/14) and group C (non-activity ^125^I seed) at 28.5% (4/14) (*P* <0.05, group A vs. B) (Figure 5B). Consistent with results mentioned above, p-MDM2-positive expression was reduced in group A (14.2%, 2/14) compared with group B (21.4%, 3/14) and group C (28.5%, 4/14) (*P* > 0.05, group A vs. B) (Figure 5C). Correlation analyses showed the following results: p-p38 and p-p53, *r* = 0.34, *P* = 0.008; p-p38 and p-MDM2, *r* = −0.42, *P* = 0.012; and p-MDM2 and p-p53, *r* = −0.38, *P* = 0.016.

## Discussion

Multiple clinical studies demonstrated the safety and efficacy of ^125^I seed implantation brachytherapy (15–17). ^125^I seed brachytherapy is impressive, especially in local tumor control and improvements in patients' quality of life. Relevant studies have confirmed the good local control rate of ^125^I seed in locally advanced non-small cell lung cancer, and compared with external irradiation in patients with lung cancer bone metastasis, the quality of life scores of ^125^I seed group are better than the external irradiation group. In the treatment of pelvic bone tumor, ^125^I seed significantly reduced bone tumor size, relieved pain, and improved the quality of life of patients, with a low complication rate (6, 11, 18).

**Figure 6:**
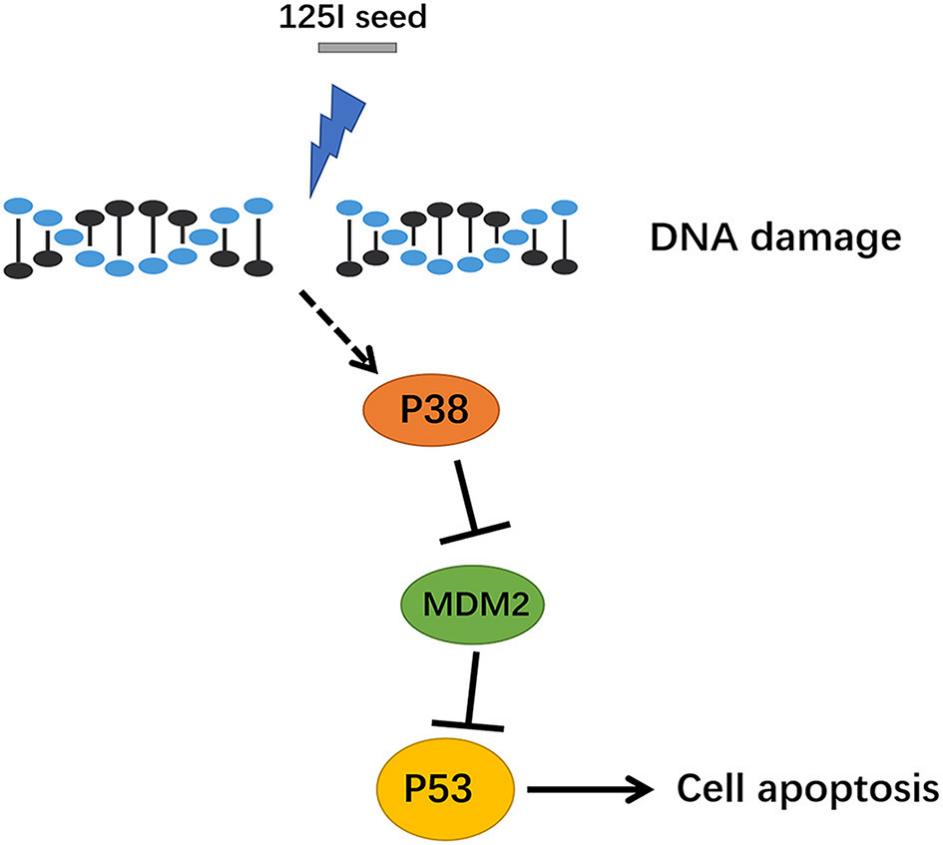
The signal path diagram of p38 MAPK/MDM2/p53. After treatment with ^125^I seed, MDM2 was downregulated, and the combination of MDM2 and p53 was reduced, which resulted in a stable transcription of p53. The p53, which has transcription activity, went into the nucleus *via* the activation of downstream apoptotic proteins, which eventually contributed to apoptosis.

Compared with the extensive development of clinical applications, research on antitumor molecular mechanisms are relatively insufficient in ^125^I seed brachytherapy. An *in vitro* study showed that altered signal transduction, activation of the Bcl-2/Bax apoptosis-related genes, and induction of cell apoptosis in gastric cancer cells after ^125^I seed irradiation (19). The present study identified MDM2 as a differentially expressed gene and found that p53 and MAPK signaling pathway expression were increased in A549 cells after 72 h of ^125^I seed irradiation. To confirm the gene chip results, MAPK phosphoprotein chips were used to detect changes in protein phosphorylation levels in A549 cells after ^125^I seed irradiation. Eleven proteins had significantly increased phosphorylation levels, including cell proliferation-related Akt, Erk1, and Erk2 and apoptosis-related p53, Jnk1, Jnk2, MKK6, p38α, p38β, p38δ, and p38γ. We hypothesized that the ^125^I seed activated the intracellular MAPK signal pathways *via* a regulatory protein and eventually activated p53 signaling pathways in the cell nucleus (Figure 6). Momand et al. first confirmed the interaction between MDM2 and p53 loci using precipitation, which means that MDM2 may be combined with the p53 protein N-terminal transcriptionally active region (20). MDM2 inhibits the p53 tumor suppressor *via* two mechanisms: ubiquitin-mediated degradation of the p53 protein and inhibition of p53 transcriptional activity, which maintains low levels of steady-state p53 in its transcriptionally inactive form (21). A negative adjustment loop exists between these proteins, which maintains MDM2 at a low level in normal cells and higher expression in tumor cells, which activates a negative feedback control loop to restrain the activity of p53 protein and affect apoptosis (22). Our study identified MDM2 as a downregulated differentially expressed genes in ^125^I seed-irradiated cells. Therefore, we hypothesized that MDM2 was a regulatory factor between the p38 MAPK signaling pathway and p53 signaling pathways, which are involved in the regulation of cell growth inhibition of ^125^I seed exposure. Previous studies detected MDM2 expression in tumor tissue and normal lung tissue in patients with NSCLC and found that MDM2 expression was related to tumor differentiation degree, clinical stage and lymph node metastasis. MDM2 protein expression may also be used to evaluate the malignancy grade and prognosis of patients with lung cancer (23). Another study found the MDM2 protein-positive expression rate was 57.8% (26/45) in lung adenocarcinoma tissues, and negative expression was found in normal tissue adjacent to tumor (*P* <0.01) (24). These studies demonstrate the important role of MDM2 in tumor development. Li et al. reported that p38 MAPK and ERK phosphorylated p53 and subsequently dissociated with MDM2 in selenite-induced apoptosis of NB4 cells (25). Another study showed that long-term low-dose radiation activated the INK4a/ARF locus in renal cell carcinomas *via* the upregulation of p38 MAPK (26). Li et al. found ^125^I combined with GEM induced stronger antiproliferation effect than single-treatment in advanced pancreatic cancer, due to the cell cycle arrest and more cellular apoptosis in PANC-1 cells. It increased Bax/Bcl-2 ratio to enhanced apoptosis (27).

The p38 inhibitor and MDM2 agonist demonstrated that the p38 MAPK/MDM2/p53 signaling pathway was involved in ^125^I seed irradiation-induced apoptosis. Park et al. reported that the p38MAPK/MDM2/p53 signaling pathway played an important role in the drug resistance mechanism (28). They found that p38 MAPK prevented MDM2 ubiquitin and p53 degradation, which increased EGFR expression and resulted in drug resistance of lung cancer cells to paclitaxel. These findings of the negative regulation relationship of p38 MAPK/MDM2/p53 is similar to our study.

In conclusion, ^125^I seed irradiation activated the p38 MAPK/MDM2/p53 signaling pathway to promote apoptosis in cells of non-small cell lung cancer. Future studies targeting this signal pathway may provide a new potential therapeutic approach for non-small cell lung cancer.

## Data Availability Statement

The datasets presented in this study can be found in online repositories. The names of the repository/repositories and accession number(s) can be found below: Gene Expression Omnibus, accession no: GSE159431.

## Ethics Statement

The animal study was reviewed and approved by the ethical standards of the Sun Yat-sen University Ethics Committees.

## Author Contributions

TZ and ZM contributed to study design and implementation. TZ, ZM, GD, and ML contributed to contributed to analysis and interpretation of data and draft of the manuscript. RT contributed to statistical analysis. FZ and ML supervised and oversaw the study. The corresponding authors had full access to all of the data and take full responsibility for the veracity of the data and the statistical analyses. All authors contributed to review and critical revision of the manuscript and approved the final version of the manuscript.

## Conflict of Interest

The authors declare that the research was conducted in the absence of any commercial or financial relationships that could be construed as a potential conflict of interest.
