# Supplementary figures and images for: 125I Seed Promotes Apoptosis in Non-small Lung Cancer Cells via the p38 MAPK-MDM2-p53 Signaling Pathway
Source: Front Oncol. 2021 Apr 21;11:582511. doi: 10.3389/fonc.2021.582511 (PMC8096899; doi:10.3389/fonc.2021.582511)

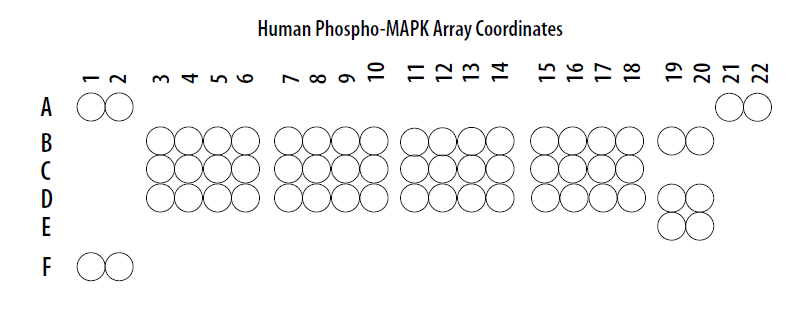

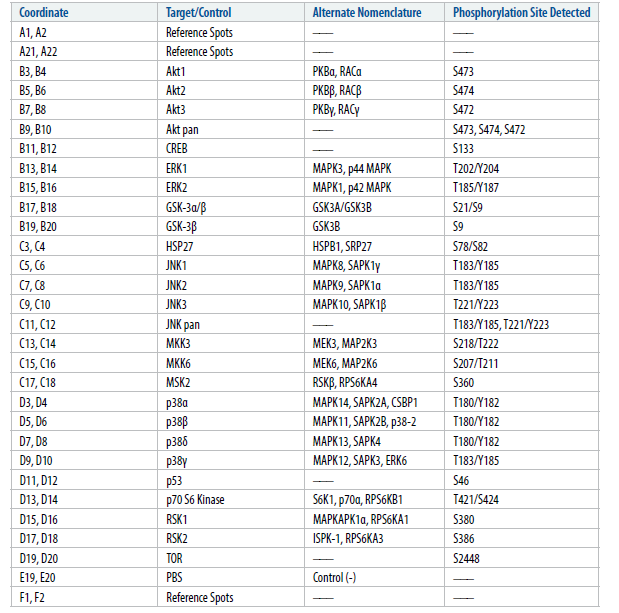

Supplement: Supplementary file 1 [file Table_1.DOCX]
